# Next-Generation Sequencing Methods to Determine the Accuracy of Retroviral Reverse Transcriptases: Advantages and Limitations

**DOI:** 10.3390/v17020173

**Published:** 2025-01-26

**Authors:** Javier Martínez del Río, Luis Menéndez-Arias

**Affiliations:** Centro de Biología Molecular Severo Ochoa, Consejo Superior de Investigaciones Científicas & Universidad Autónoma de Madrid, c/Nicolás Cabrera 1, 28049 Madrid, Spain

**Keywords:** reverse transcriptase, next-generation sequencing, fidelity of DNA synthesis, retrovirus, cDNA synthesis

## Abstract

Retroviruses, like other RNA viruses, mutate at very high rates and exist as genetically heterogeneous populations. The error-prone activity of viral reverse transcriptase (RT) is largely responsible for the observed variability, most notably in HIV-1. In addition, RTs are widely used in biotechnology to detect RNAs and to clone expressed genes, among many other applications. The fidelity of retroviral RTs has been traditionally analyzed using enzymatic (gel-based) or reporter-based assays. However, these methods are laborious and have important limitations. The development of next-generation sequencing (NGS) technologies opened the possibility of obtaining reverse transcription error rates from a large number of sequences, although appropriate protocols had to be developed. In this review, we summarize the developments in this field that allowed the determination of RNA-dependent DNA synthesis error rates for different RTs (viral and non-viral), including methods such as PRIMER IDs, REP-SEQ, ARC-SEQ, CIR-SEQ, SMRT-SEQ and ROLL-SEQ. Their advantages and limitations are discussed. Complementary DNA (cDNA) synthesis error rates obtained in different studies, using RTs and RNAs of diverse origins, are presented and compared. Future improvements in methodological pipelines will be needed for the precise identification of mutations in the RNA template, including modified bases.

## 1. Introduction

RNA viruses replicate as complex distributions of closely related variant genomes known as quasispecies [1]. These mutant swarms are subjected to genetic variation, competition and selection, and their diversity is fueled by the high mutation rates of RNA viruses. Genetic variability is one of the hallmarks of human immunodeficiency virus (HIV) infection. The large diversity of HIV is a consequence of its high mutation and recombination rates, the high replication rate of the virus leading to large progenies, and to selective forces that operate during viral dissemination (e.g., immune pressure, host factors, etc.). Estimated mutation rates for HIV type 1 (HIV-1) obtained from studies involving within-host longitudinal samples of DNA sequences could be as high as 10^−3^ nucleotide substitutions per site per year [2,3,4], although these rates could be different depending on the specific genomic region analyzed [5].

The genome of HIV is composed of two copies of non-covalently linked, unspliced, positive-sense single-stranded RNA (ssRNA). The viral genomic RNA molecules resemble messenger RNA, bearing a 5′ cap (Gppp) and a 3′ poly(A) tail. Each RNA has a length of approximately 10 kB and contains several open reading frames, including the characteristic retroviral genes *gag*, *pol* and *env*. These genes encode polyproteins Gag, Pol and Env that, after cleavage, produce structural proteins, viral enzymes and envelope glycoprotein, respectively.

A reverse transcriptase (RT) encoded within the *pol* gene is responsible for the conversion of the genomic ssRNA into double-stranded DNA (dsDNA) that integrates into the host’s chromosome. This process, known as reverse transcription, is relatively complex (for reviews, see [6,7]) and requires the participation of the RNA- and DNA-dependent DNA polymerase and ribonuclease H (RNase H) activities of the RT. Unlike cellular DNA polymerases, retroviral RTs are devoid of 3′→5′ exonucleolytic proofreading activity and are therefore less accurate than prokaryote and eukaryote DNA polymerases while synthesizing DNA (for reviews, see [8,9,10]). Although retroviral variation is largely due to errors made by the RT during reverse transcription, cellular RNA polymerase II and a series of viral and host factors also contribute to the retroviral mutation rate [11]. Unbalanced nucleotide pools inside the infected cells can facilitate the emergence of specific mutations [12]. More importantly, cytidine deaminases of the APOBEC (apolipoprotein B mRNA editing enzyme, catalytic polypeptide) family, such as APOBEC3F and APOBEC3G, are potent inhibitors of HIV-1 infection, while promoting hypermutation G→A in plus-stranded cDNA during reverse transcription [13]. On the other hand, adenosine deaminases acting on RNA (ADAR) play a dynamic and nuanced role in regulating transcriptomes and are expected to contribute to increase viral diversity, as demonstrated in different RNA viruses, including HIV [14,15].

Studies on the fidelity of RTs have been justified by the importance of HIV-1 RT as a target of antiretroviral therapy [16], as well as for the pivotal role of retroviral enzymes in biotechnological applications, from conventional RT-PCR to transcriptomics and prime editing [17]. The accuracy of DNA synthesis catalyzed by purified RTs has traditionally been determined using enzymological (gel-based) assays or by measuring mutant frequencies in a reporter gene (typically *lacZ*α in forward mutation assays) (reviewed in [9,10]). However, these methods are laborious and time-consuming.

The introduction of next-generation sequencing (NGS) technologies has facilitated the analysis of a large number of sequences and the detection of many mutations (as well as minority variants) resulting from single experiments. However, these techniques have some important limitations resulting from their inherent mutational background. Improvements in the methodological workflow, such as, for example, the use of barcodes or unique molecular identifiers (UMIs), can be helpful to extract relevant data on the accuracy of reverse transcription in vitro. In this review, we describe different approaches and NGS-based methods to determine the fidelity of RTs and discuss their advantages and limitations.

## 2. RT Structure

**Figure 1:**
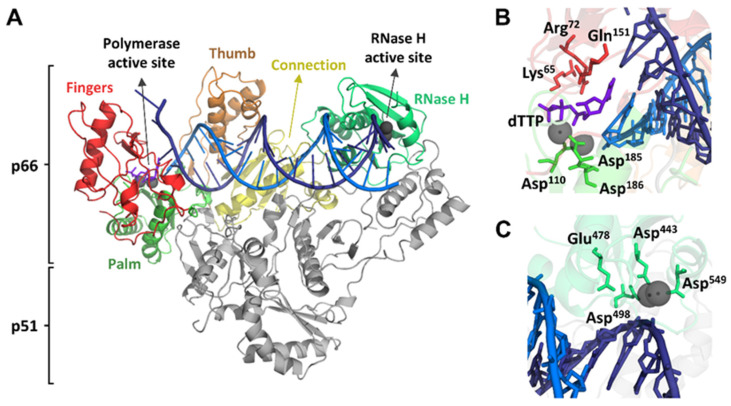
HIV-1 RT structure. (**A**) Crystal structure of HIV-1 RT bound to a DNA primer-template and an incoming deoxythymidine triphosphate (dTTP) (PDB ID: 1RTD). Subdomains of the p66 DNA polymerase domain are shown as red (fingers), green (palm), orange (thumb) and yellow (connection). The RNase H domain is shown as mint green. The p51 subunit is colored gray. The template strand is represented in dark blue, the primer strand in light blue, and the incoming dTTP is shown as purple. Coordinating metal ions (Mg^2+^) in the DNA polymerase and RNase H active sites are represented as dark gray spheres. (**B**) Detailed view of the DNA polymerase active site, showing the location of the catalytic triad Asp110, Asp185 and Asp186, along with key residues Lys65, Arg72 and Gln151, the incoming dTTP, the template-primer and the two metal ions. (**C**) Detailed view of the RNase H active site, showing the location of Asp443, Glu478, Asp498 and Asp549 (DEDD motif) interacting with the metal ions and the template-primer.

Retroviral RTs are structurally diverse. The best characterized enzyme is the HIV-1 RT. Currently, the number of crystal structures of this enzyme deposited in the Protein Data Bank is close to 200 and includes complexes of wild-type and mutant RTs with selected inhibitors, as well as binary (RT-DNA/DNA and RT-RNA/DNA) and ternary complexes (e.g., RT-DNA/DNA-dNTP) containing different types of nucleic acids (for a recent review, see [18]). The HIV-1 RT is an asymmetric heterodimer composed of subunits of 66 and 51 kDa, termed as p66 and p51, respectively (Figure 1). The p66 subunit contains a DNA polymerase domain spanning residues 1–440 and an RNase H domain including residues 441–560. Catalytic sites of the DNA polymerase and RNase H activities are located in p66. Active DNA polymerase residues are Asp110, Asp185 and Asp186, while the catalytic residues responsible for the cleavage of RNA in RNA/DNA complexes are Asp444, Glu478, Asp498 and Asp549. Both catalytic sites coordinate divalent cations (Mg^2+^ in physiological conditions, although Mn^2+^ binding can be observed in some structures). The p51 subunit has the same amino acid sequence as p66 but lacks the RNase H domain and has a structural role while contributing to enzyme dimerization [19].

As expected, similar crystal structures have been determined for closely related RTs, such as those of HIV-2 [20] or feline immunodeficiency virus (FIV) [21]. Similar to lentiviral RTs, Baldwin et al. [22] recently reported the crystal structure of a ternary complex containing the human endogenous retrovirus-K (HERV-K) RT bound to double-stranded DNA and an incoming dNTP. Its structure shows a striking similarity to the HIV-1 RT asymmetric heterodimer.

Other examples of heterodimeric retroviral RTs are those of alpharetroviruses, such as Rous sarcoma virus (RSV), avian leucosis virus (ALV) or avian myeloblastosis virus (AMV). AMV RT is a heterodimer consisting of a 63-kDa α subunit and a 95-kDa β subunit. The α subunit contains the DNA polymerase and RNase H domains of RT, while the β subunit has an additional C-terminal integrase domain [23].

In contrast, murine leukemia virus (MLV) RT is a monomer of 80 kDa. Wild-type and recombinant variants of MLV RT, particularly those showing high thermal stability, are probably the most frequently used enzymes in applications requiring the synthesis of complementary DNA (cDNA) [17]. The crystal structure of the closely related xenotropic murine leukemia virus-related virus (XMRV) RT complexed with double-stranded DNA has been reported [24]. Interestingly, studies carried out with marmoset foamy virus (MFV) RT have revealed an unexpected ability of this enzyme to adopt monomeric or dimeric configurations, depending on the type of bound substrate [25]. Thus, MFV can adopt both a monomeric configuration in the presence of RNA/DNA hybrids and an asymmetric dimer arrangement in the presence of dsDNA. Foamy virus RTs are enzymes of around 750 residues with all the enzymatic functions of the viral protease and RT. The enzyme has an N-terminal protease domain bound to the RT and is monomeric in solution [26,27].

Recently, interest has been focused on prokaryotic RTs, which are extraordinarily diverse and are key components of different lines of defense against phages and other mobile elements (for a recent review, see [28,29]). All these enzymes have enormous biotechnological potential. Among them, thermostable intron II RTs, such as TGIRT^®^-III RT, MarathonRT or Induro^®^ RT, are commercialized and used in many transcriptomic applications. TGIRT^®^-III RT is a group II intron RT purified from *Geobacillus stearothermophilus* [30], while MarathonRT was obtained from *Eubacterium rectale* [31]. These enzymes are all thermostable and show better performance than engineered thermostable MLV RTs while copying transcripts longer than 10 kb. The crystal structure of a full-length thermostable group II intron RT (TGIRT^®^-III RT) in complex with an RNA/DNA hybrid and incoming dNTP has been obtained at 3.0-Å resolution [32]. The structure is similar to those of retroviral RTs, but unlike HIV-1 RT, the group II intron RT binds the template-primer substrate as a monomer.

## 3. Methods to Determine the Fidelity of DNA Synthesis Catalyzed by Purified RTs

Soon after the discovery of retroviral RTs in 1970, and taking advantage of research focused on the effects of potential carcinogens in promoting mutagenesis in DNA polymerization, researchers developed biochemical assays measuring the simultaneous incorporation of complementary and non-complementary nucleotides during DNA synthesis catalyzed by purified RTs. These classical assays included measurements of the incorporation of dCMP in place of dTMP in cell-free DNA synthesis with polyadenylic acid as a template or competition assays between complementary dGTP and noncomplementary dATP, using poly(rC)-oligo(dG) as a template-primer, and allowed estimates of fidelity for AMV RT [33,34]. Further sophistication of the assays led to the development of enzymological or “gel-based” assays that provided accurate measurements of the nucleotide selectivity of the polymerase at a given position, although their analysis was limited to a relatively small number of incorporation sites. Later on, researchers developed genetic assays using reporter genes that allowed the exploration of a wide variety of sequence contexts and helped with the identification of relevant hot spots related to RT errors. These methods are briefly summarized in the following sections.

### 3.1. Enzymological Assays

Enzymological assays include dNTP exclusion assays, misinsertion assays and mismatch extension assays. The dNTP exclusion assays, also known as “minus” sequencing gel assays or primer extension assays, provide a rough estimate of enzyme fidelity [35]. In these assays, only three dNTPs are supplied to extend an oligonucleotide primer annealed to a template. A faithful polymerase is expected to halt synthesis at the position where the missing dNTP would normally be incorporated. Therefore, the presence of full-extension bands indicates the insertion of incorrect nucleotides. The fidelity of RTs can be compared by examining the pattern of bands produced by each enzyme.

Quantitative characterization of nucleotide incorporation kinetics by RTs and other DNA polymerases has been used to analyze nucleotide selectivity in different sequence contexts and with many different wild-type and mutant polymerases (for reviews, see [8,36]). In misinsertion fidelity assays, the ability to extend a primer is evaluated in the presence of correct or incorrect nucleotides using a template-primer. The efficiency of primer elongation is measured through quantitative gel electrophoresis, and the resulting data are analyzed using the polymerase kinetic parameters *k*_pol_ and *K*_D_ for each nucleotide [37,38]. Misinsertion efficiency (*f*_ins_) is calculated as the ratio of *k*_pol_/*K*_D_ for the incorrect nucleotide to that for the correct nucleotide:

*f*_ins_ = *k*_pol_/*K*_D_ (incorrect nucleotide)/*k*_pol_/*K*_D_ (correct nucleotide)

On the other hand, mismatch extension assays evaluate DNA polymerase accuracy by comparing the kinetics of extending template-primer complexes with a mismatched 3′-end primer (e.g., C-C) and with a correctly matched template-primer hybrid (e.g., G-C). The mismatch extension ratio (*f*_ext_) is calculated by dividing the catalytic efficiency (*k*_pol_/*K*_D_) of the mismatched primer by that of the correctly matched primer:

*f*_ext_ = *k*_pol_/*K*_D_ (mismatched primer)/*k*_pol_/*K*_D_ (matched primer)

Although *k*_pol_ and *K*_D_ are the preferred constants, their measurement requires rapid transient kinetics and sophisticated equipment for their correct assessment. Alternatively, equivalent parameters (i.e., *k*_cat_ and *K*_M_) can be obtained under steady-state conditions, although in this case, the obtained values are strongly influenced by the slow rate of dissociation of the template-primer from the enzyme (*k*_off_). In general, enzymological assays offer precise measurements of nucleotide selectivity at specific positions. However, they are labor-intensive, limited to a small number of incorporation sites and, for precise determinations, sophisticated equipment (quench-flow apparatus) might be needed. These methods provide good estimates of nucleotide selectivity, but the obtained results cannot be interpreted in terms of an overall error rate [8].

### 3.2. Genetic Assays

Genetic assays estimate fidelity by measuring mutant frequencies in a reporter gene (e.g., *lacZ*α, *gfp*, *htk*) (for reviews see [8,9]). The assays can be designed to detect the reversion of an inactivating mutation introduced in the reporter gene (codon reversion assays) or to detect the inactivation of the reporter gene (forward mutation assays). Codon reversion assays measure base substitution rates of DNA polymerases using single-stranded phage DNA templates with nonsense codons, such as amber (TAG) in phage ΦX174 or opal (TGA) in the *lacZ* gene of bacteriophage M13mp2 (reviewed in [9]). Reversion of these codons to coding triplets is detected as revertant plaques after bacterial transformation or as dark blue M13 plaques on X-Gal indicator plates. These assays typically focus on specific base substitution errors, particularly G→A transitions. However, the limited scope of target sites may not accurately reflect the overall mutation rate.

**Figure 2:**
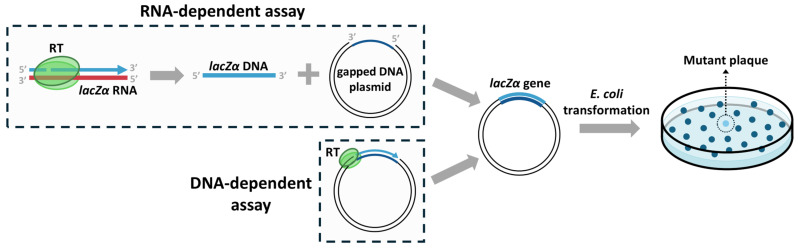
Overview of forward mutation assays with the *lacZ*α gene. The initial steps (outlined with dashed lines) differ depending on the desired measurement. For RNA-dependent DNA polymerase fidelity assessment, the RT uses an RNA molecule with the *lacZ*α sequence as a template, and the obtained DNA is hybridized with a gapped DNA plasmid carrying the complementary sequence of the *lacZ*α gene. In the case of DNA-dependent DNA polymerase fidelity, the gapped DNA plasmid serves as template for the RT to produce the complementary *lacZ*α gene strand. Following the initial steps, *E. coli* is transformed with the resulting plasmids, and errors are identified as light blue or colorless colonies.

Forward mutation assays assess the fidelity of RTs by detecting nucleotide changes across many sites within a reporter gene [39]. This method typically involves the use of a gapped dsDNA template from phage M13mp2, where the *lacZ*α gene in one of the two strands is missing. The gapped DNA serves as template for DNA-dependent DNA synthesis to obtain the complementary strand of the *lacZ*α gene (the antisense strand of the gene). The resulting DNA is then used to transform *Escherichia coli* cells, which are then plated on X-Gal indicator plates. Mutations can be identified by altered plaque color, such as light blue or colorless, indicating a loss of β-galactosidase activity because of errors introduced by the RT. If desired, DNA from mutant plaques can be purified and sequenced by Sanger sequencing to determine the specific mutations introduced. The method can also be adapted to study RNA dependent DNA polymerase fidelity (Figure 2).

Forward mutation assays offer several advantages, such as the ability to detect a wide range of mutations, including base substitutions and frameshifts, across many sites, making them valuable for identifying mutational hotspots. However, they have limitations (for reviews, see Refs. [8,9,36]). First, *Escherichia coli* mismatch repair machinery can potentially correct some errors. Secondly, even if the errors are not corrected, not all mutations result in detectable phenotypic changes, especially synonymous mutations that do not alter the protein sequence. A third limitation is that the observed mutation rates represent the sum of the RT mutation rate and errors already present in the template, especially relevant in the RNA-dependent assays. Additionally, the method can be labor-intensive and subjective, as plaque color changes may be subtle.

## 4. NGS-Based Methods for RT Fidelity Measurements

Over the past two decades, the advent of NGS technologies has revolutionized the ability to assess error rates in a relatively short time and using vast amounts of data [40,41]. However, short-read NGS platforms (e.g., Illumina), exhibit background error rates around 10^−2^ mutations per site and 10^−3^ to 10^−4^ mutations per site for stringently filtered data [42,43,44,45], too high for a reliable determination of DNA polymerase error rates. On the other hand, long-read NGS technologies (also referred to as third-generation sequencing technologies), such as PacBio or Oxford Nanopore, show even higher error rates, reaching up to 10^−1^ errors per base [46].

High error rates in sequencing create background noise that hinders the measurement of RT errors, which are typically below 10^−4^ in forward mutation assays [8,9]. Additionally, certain steps during library preparation (e.g., PCR amplification or gel electrophoresis) contribute to background errors. Consequently, accurate determinations of RT error rates require strategies to minimize and distinguish those artifacts.

The most widely used strategy for artifact correction is “consensus sequencing”. This approach identifies and eliminates artifacts introduced by sequencing and during library preparation steps. It is valuable not only for determining RT error rates but also for other applications, such as detecting low-frequency mutations in RNA and DNA samples (reviewed in [47]). Suitable methods based on consensus sequencing were described for the first time around 15 years ago [48,49,50].

**Figure 3:**
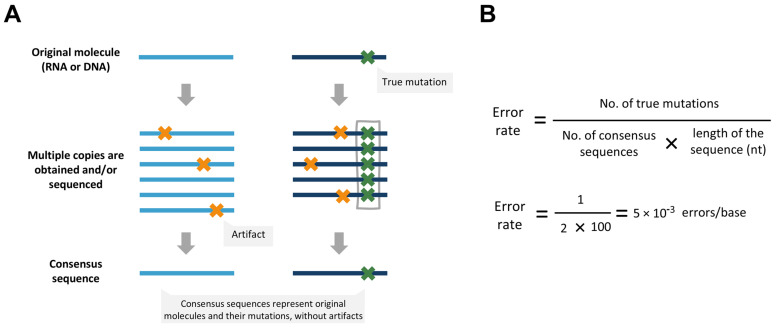
Rationale of consensus sequencing. (**A**) Error correction of consensus sequencing. Depending on the method, multiple copies of an original molecule (DNA or RNA) are obtained and sequenced, or the molecule is sequenced several times. Sequences that derive from the same molecule are aligned, and a consensus is established. True mutations from the original molecule, which are expected to be present in all copies, are added to the consensus. On the other hand, artifacts, which are expected to be inconsistent across the copies, are discarded. (**B**) Formula to calculate error rates. Once consensus sequences are obtained, an accurate error rate can be calculated. If we consider that the consensus sequences shown on the left have a length of 100 nt, the error rate of the original molecules would be 5 × 10^−3^ errors/base.

Consensus sequencing can be approached using different strategies, all sharing the common principle of obtaining multiple sequences that derive from an original DNA or RNA molecule (Figure 3). To analyze errors introduced by RTs, multiple copies of the same original cDNA molecule are typically examined. Mutations consistently observed at the same position across all molecules are considered true errors introduced during reverse transcription, whereas mutations appearing only in some molecules are regarded as sequencing errors or other artifacts.

A number of consensus generation methods have been used (or could potentially be used) to measure RT errors. These methods can be classified depending on the use of barcodes.

### 4.1. Methods Based on the Use of Barcodes

A widely adopted approach for generating consensus sequences in short-read sequencing is the use of barcodes or UMIs, which are sequences of random degenerate nucleotides (typically > 8 nucleotides in length). These barcodes can be added to RNA molecules used as templates for reverse transcription, cDNA molecules obtained or both types of molecules.

**Figure 4:**
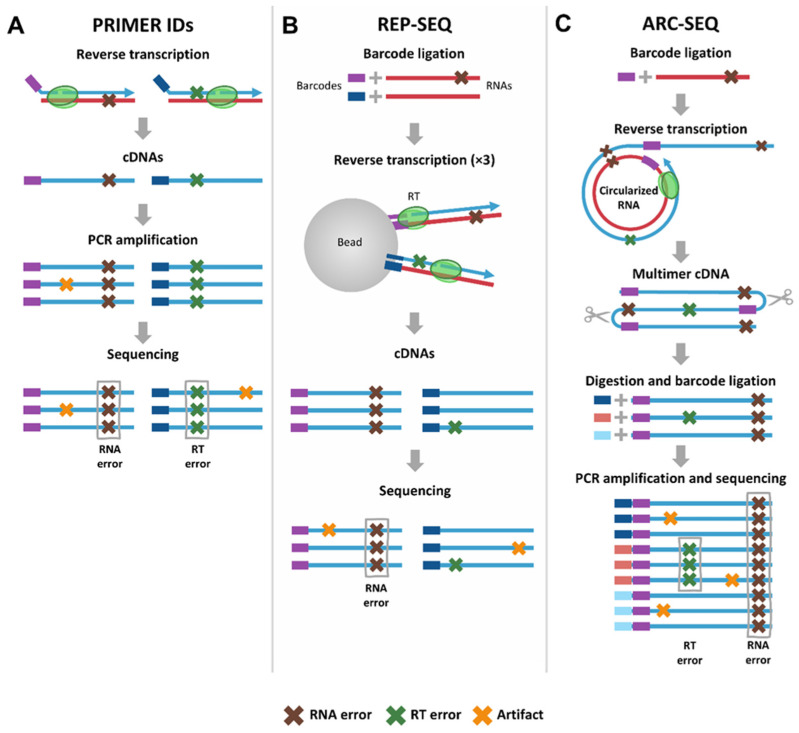
Barcode-based methods for reverse transcription error rate measurements. Brown crosses represent RNA errors, green crosses depict RT errors and yellow crosses represent artifacts (i.e., other errors, such as sequencing or PCR errors). (**A**) PRIMER IDS [49]. Reverse transcription is performed using primers with barcodes at their 5′ ends. The labeled cDNAs are amplified by PCR and sequenced. Combined RNA and reverse transcription errors are expected to be in all sequences sharing a barcode. (**B**) Replicated sequencing (REP-SEQ) [57]. RNA molecules are tagged with barcodes and attached to beads. Reverse transcription is performed, and cDNAs are washed away multiple times. The resulting cDNAs are then sequenced. Transcription errors are expected to be present in all reads sharing a barcode and are distinguished from RT errors and artifacts. (**C**) Accurate RNA consensus sequencing (ARC-SEQ) [58]. Barcoded RNA molecules are circularized and subjected to rolling circle reverse transcription, yielding tandem-repeated cDNA copies. These multimeric cDNAs are restricted to cDNA monomers, which are then tagged with barcodes, amplified through PCR and sequenced. Transcription errors are expected to be present in all sequences sharing an RNA barcode, while reverse transcription errors should be shared only among sequences with the same cDNA barcode.

Barcoding of cDNA molecules is a fast, effective and widely used strategy, initially described as the “PRIMER IDs” method by Jabara et al. [49]. This technique was first applied to identify minority variants found in viral genomic RNA from HIV-infected patients. Later, this approach was used by several labs to identify RT errors and measure RT error rates [31,51,52,53,54,55,56]. The procedure is summarized in Figure 4A. Reverse transcription is performed to generate cDNAs using primers that incorporate a unique barcode at their 5′ end. This process renders cDNAs tagged with different barcodes and ensures that each cDNA molecule is uniquely identified. The barcoded cDNAs are then amplified via PCR, followed by sequencing. Reads sharing the same UMI are grouped together, allowing for the identification of mutations that are consistently observed within each group. These mutations are considered to be original errors present in the cDNA molecule, as they are likely to have arisen during the transcription or reverse transcription processes.

Another approach similar to the PRIMER IDs method is known as duplex sequencing, or DUP-SEQ [59]. Unlike the PRIMER IDs method, which generates a consensus sequence from a single DNA strand, DUP-SEQ creates consensus sequences for each strand of a dsDNA molecule. In this method, following reverse transcription, the complementary strand of the cDNA is synthesized, and special adapters containing barcodes are added to both ends of the dsDNA. These adapters individually label each strand and enable their independent amplification due to the presence of non-complementary segments in their structure. Finally, the amplified copies are sequenced and analyzed in silico. This process allows for the generation of separate consensus sequences for both the original cDNA strand and its complementary strand. True mutations are expected to be found in the same position in both sequences, while mutations present in only one strand would be considered artifacts not discarded during the single-strand consensus construction. While duplex sequencing methods have been widely used to study ultra-rare mutations in DNA samples [59,60,61], they have not yet been applied to measure RT fidelity. Nevertheless, this approach could prove valuable for studying high-fidelity RTs or identifying errors during second-strand DNA synthesis.

Both the PRIMER IDs and DUP-SEQ methods provide a robust framework for distinguishing true mutations from errors introduced during sequencing or PCR amplification. However, these mutations reflect not only errors introduced by the RT used but also mutations present in the RNA template. Consequently, in these setups, it is not possible to distinguish between RNA-derived mutations and reverse transcription errors.

The application of barcodes to RNA molecules allows the identification of RNA mutations. For example, Gout et al. [57] identified RNA mutations with a method called REP-SEQ (Figure 4B). In REP-SEQ procedures, RNA molecules are tagged with barcodes and bound to beads. Reverse transcription is carried out, followed by repeated washing of the cDNAs. The resulting cDNAs are then sequenced. Transcription errors are expected to appear in all reads sharing the same barcode. RT errors can then be estimated as those mutations not attributable to the RNA, but this estimation may also include artifacts introduced during library preparation.

The best way to discard both RNA mutations and sequencing artifacts would be the labeling of both RNA and cDNA, as in the ARC-SEQ method [58] (Figure 4C). This dual-barcoding approach enables researchers to distinguish between RNA-derived mutations and errors introduced during reverse transcription and, at the same time, allows the identification and elimination of sequencing artifacts. RNA molecules are first tagged with barcodes, circularized and subjected to rolling circle reverse transcription, a process that generates tandem repeats of cDNA copies. These multimeric cDNAs are then processed into individual cDNA monomers, each of which is then tagged with a unique barcode. The barcoded cDNAs are amplified through PCR and subsequently sequenced. In this method, transcription errors are expected to be present across all sequences that share the same RNA barcode, as these errors occur during the transcription of the RNA itself. On the other hand, reverse transcription errors should only appear in sequences that share the same cDNA barcode. This method has been exclusively applied to analyze RNA errors in published studies, but it could be adapted to focus on RT errors as well.

### 4.2. Methods Avoiding the Use of Barcodes

While the use of RNA barcodes in conjunction with cDNA barcodes could effectively identify reverse transcription errors, adding barcodes to RNA presents significant challenges. Indeed, Traverse and Ochman [62] reported difficulties in reproducing results obtained with the REP-SEQ method. In view of these problems, most studies assessing the fidelity of RTs have either employed the “PRIMER IDs” approach or resorted to barcode-less alternatives.

**Figure 5:**
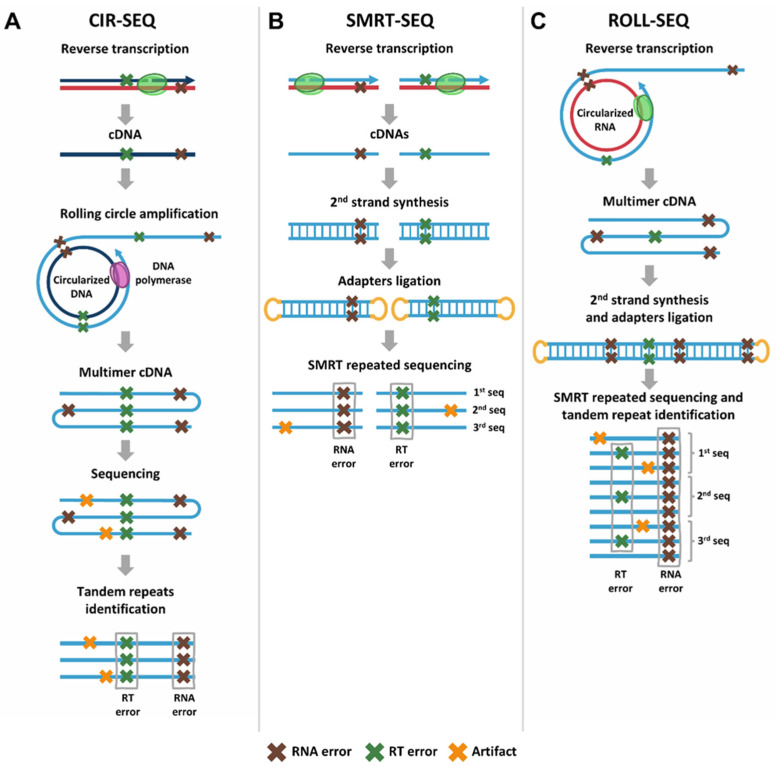
Barcode-free methods for reverse transcription error rate measurements. Brown crosses represent RNA errors, green crosses depict RT errors and yellow crosses represent artifacts (i.e., other errors, such as sequencing or PCR errors). (**A**) Circular sequencing (CIR-SEQ) [63]. Circularized DNA fragments (for example, cDNAs obtained after reverse transcription) are obtained and used as a template for rolling circle amplification reactions to obtain multimer DNAs. After sequencing, the repeated units of each multimer cDNA are identified and aligned. Transcription errors are expected to be found in all the aligned sequences from the same multimer cDNA molecule. (**B**) Single molecule real-time sequencing (SMRT-SEQ) [64]. RNA is used as template for reverse transcription. The obtained cDNAs are used as templates for second-strand DNA synthesis. PacBio SMRT adapters are attached to the dsDNA and subjected to SMRT repeated sequencing. Each read of the original cDNA strand is aligned. Combined RNA transcription and reverse transcription errors are expected to be in all the aligned sequences. Additionally, second-strand DNA synthesis errors could be identified by aligning the reads of the complementary strand. (**C**) Rolling circle sequencing (ROLL-SEQ) [65]. Rolling circle reverse transcription reactions are conducted to obtain multimeric cDNAs. The complementary DNA strand is then synthesized, and PacBio SMRT adapters are attached. SMRT repeated sequencing is performed, and the repeated monomers of the cDNA strand are identified for each read. RNA errors are expected to be in all the monomers of the same read and in all reads, while RT errors are expected to be only in the aligned reads of the same monomer.

A straightforward example of a barcode-less method is CIR-SEQ [63] (Figure 5A), which utilizes rolling circle amplification. This process is conceptually similar to the rolling circle reverse transcription used in the ARC-SEQ method. In CIR-SEQ, DNA templates (which may be generated after reverse transcription of RNA molecules) are circularized. These circularized templates are then amplified multiple times in tandem using a rolling circle polymerase, producing long, repetitive sequences. These tandem-repeat molecules are subsequently sequenced, and the resulting sequences are analyzed to derive a consensus sequence. To date, this method has primarily been used to measure mutations in DNA, but it could also be adapted for the determination of RT error rates.

However, as found in the case of the “PRIMER IDs” method, error rates obtained with CIR-SEQ would reflect the cumulative errors introduced during the reverse transcription process. The CIR-SEQ method has also been adapted to study RNA mutations [66,67,68,69]. In those adaptations (also named CIRC-SEQ), tandem-repeat cDNAs are directly obtained from RNA molecules via rolling circle reverse transcriptions. These multimeric cDNAs are sequenced and are then analyzed the same way as described above, but mutations identified in consensus sequences will only represent RNA errors.

Another barcode-free consensus generation method is SMRT-SEQ [70], a long-read sequencing technology that allows repeated sequencing of the same DNA molecule. This method was used by Potapov et al. [64] to measure the error rates of several RTs (Figure 5B). In these analyses, RNA serves as a template for reverse transcription, generating cDNA molecules. The resulting cDNAs are then used as templates for second-strand DNA synthesis, producing double-stranded DNA. PacBio SMRT adapters are attached to the dsDNA, which is then subjected to repeated sequencing using SMRT technology. Each read corresponds to the original cDNA strand, and these reads are aligned to generate a consensus sequence. Like cDNA barcoding methods, SMRT-seq captures both reverse transcription errors and RNA-derived errors, making it challenging to distinguish between the two types of errors.

A recently published method, ROLL-SEQ [65] (Figure 5C), combines elements of CIR-SEQ and SMRT-SEQ: it incorporates rolling circle reverse transcription while allowing differentiation of RNA errors. This method provides a more accurate measurement of RT errors by distinguishing them from the RNA-specific errors. In this approach, rolling circle reverse transcription reactions are performed to generate multimeric cDNA molecules. Once these multimeric cDNAs are synthesized, the complementary DNA strand is created, and PacBio SMRT adapters are attached to the dsDNA. The samples undergo SMRT repeated sequencing, which captures long reads and allows for the identification of repeated monomers of the cDNA strand in each sequencing read. RNA-derived errors are expected to appear in all monomers of the same read, as well as in all reads that share the same RNA template. In contrast, RT errors are expected to appear only in the aligned reads of the same cDNA monomer. Both methods based on SMRT sequencing (i.e., SMRT-SEQ and ROLL-SEQ) offer the potential to study second-strand DNA synthesis errors if desired. As in the case of duplex sequencing, it is possible to generate independent consensus sequences for each strand: the one corresponding to the cDNA and its complementary strand.

In addition, we can also find barcode-less error correction strategies applied to Oxford Nanopore sequencing. For example, INC-SEQ [71] combines rolling circle amplification, followed by nanopore sequencing. Unlike the ROLL-SEQ method, it only allows the sequencing of a single strand. This method has been used to detect low-abundance mutations, but it could also be used to measure errors in cDNA molecules.

## 5. Limitations of NGS-Based Fidelity Measurements

Measuring and comparing the error rates of RTs is often challenging. Even when the same type of experiment and the same RT enzyme are used, numerous factors can influence fidelity. Since RTs can synthesize DNA using two types of templates, it is important to distinguish between fidelity when using RNA or DNA as a template. Other factors that can affect the measured fidelity include the sequence of the template, the source of the template (e.g., chemically synthesized, in vitro synthesized by polymerases, extracted from biological samples), the presence of nucleic acid modifications, mutations in the RT that may affect fidelity and conditions during reverse transcription (e.g., temperature, magnesium concentration, pH, nucleotide concentration) [55,56,64,72], which are reviewed in Refs. [9,73].

### 5.1. NGS Challenges

In contrast to other methods, NGS assays enable the rapid and efficient determination of error rates across a large number of sequences and can identify all types of errors, including silent mutations. However, there is a great diversity of techniques and bioinformatics pipelines to analyze the results. The parameters employed in bioinformatics analyses vary between studies and can significantly affect error rate calculations. As a result, a consistent standard for measuring RTs’ fidelity with NGS platforms is still lacking. This also includes the bioinformatics pipelines used to analyze the NGS results and their parameters. Open and clearly documented pipelines are required to allow for the easy reproduction of results while ensuring transparency in the analysis of NGS data.

### 5.2. Barcode Challenges: Consensus Sequences and Cutoff and Threshold Values

A common feature of all NGS methods is the need to generate consensus sequences in order to identify true mutations and discard artifacts. Two key parameters are critical: the minimum number of sequences required to build a consensus sequence (cutoff) and the threshold used to identify mutations, i.e., the minimum percentage of sequences in which a mutation must be present (at a given position) to be considered a true mutation. For example, a 100% threshold requires the mutation to appear in all sequences to be included in the consensus, whereas a 50% threshold allows the mutation to be included if it appears in at least half of the sequences.

**Figure 6:**
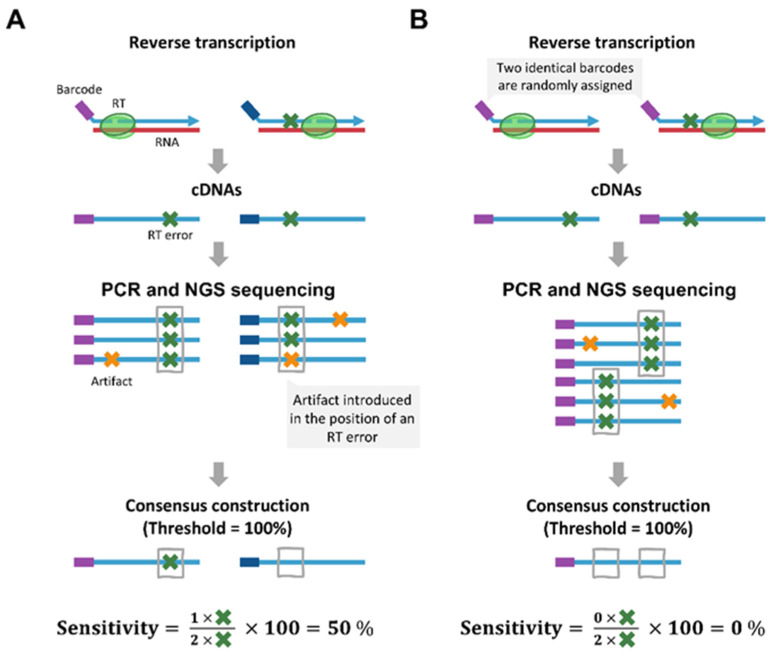
Sensitivity in cDNA barcoding methods. (**A**) shows an example where sensitivity is affected by the introduction of an artifact during PCR or sequencing at a position already containing an RT mutation. (**B**) illustrates a scenario where sensitivity is impacted by assigning the same barcode to sequences originating from different cDNAs. This can occur if barcodes with identical sequences are used by chance during reverse transcription. Alternatively, different barcodes may initially be assigned, but a mutation introduced during library preparation can generate a new barcode sequence that matches another barcode already present in the library pool. Sensitivity is calculated as the percentage of original mutations found in the consensus sequences relative to all mutations present in the original cDNAs that underwent PCR amplification and sequencing.

The choice of cutoff and threshold values can significantly affect error measurements. However, there is no consensus on the optimal parameters for accurate mutation selection, and a wide variety of parameter choices exist (discussed below). Reid-Bayliss and Loeb [58] reported that, for a fixed 70% threshold, error rates reach a plateau when using a cutoff of three. On the other hand, in the study by Martínez del Río et al. [56], errors were analyzed with different cutoffs and thresholds, as well. For instance, with a fixed cutoff of at least four sequences per consensus, the authors found a difference of one order of magnitude in the HIV-1 RT error rate when using a 0% threshold as compared to 100% threshold (1.6 × 10^−4^ and 1.6 × 10^−5^ errors/base, respectively). The study also included computational simulations to assess threshold selection, revealing a trade-off between sensitivity (also known as recall or true positive rate) in detecting true mutations and specificity (true negative rate) in discarding artifacts. Strict 100% thresholds provide high specificity at the cost of reduced sensitivity, while less stringent thresholds (0–50%) achieve 100% sensitivity but include more artifacts. Figure 6 illustrates two scenarios where sensitivity can be compromised if a high threshold is applied.

### 5.3. Strategies to Avoid Sequencing Artifacts

As mentioned before, another issue is the inclusion of artifacts as true errors (low specificity), especially when a low threshold is used to reach a high sensitivity. The repeated occurrence of an artifact in the same position of sequences could be explained by several factors. For example, if the method includes PCR amplification, errors introduced in the first steps are expected to be present in a high number of DNA molecules that are sequenced. However, sequencing pipelines involving the elimination of sequences neighboring the target sequence have been designed to reduce errors introduced in the first amplification steps, reporting a 100-fold reduction over standard Illumina error rates [74]. On the other hand, the occurrence of sequencing error hotspots could be a source of repeated artifacts. Deakin et al. [75] performed Illumina sequencing with Sanger-sequenced barcodes and reported an uneven distribution of NGS errors along the sequences, with pronounced sequencing error hotspots.

The use of accurate sequencers, stringent quality filtering and strategies like paired-end sequencing can undoubtedly reduce artifacts due to sequencing errors [76] but it does not offer a perfect solution to discard artifacts, especially considering there may also be other sources of artifacts. Including controls in experiments can help estimate background errors that may be quantified despite the consensus-building strategy. However, the use of controls is often limited. For example, Wang et al. [55], who used the cDNA barcoding method to measure HIV RT error rates, aimed to estimate background error rates by using a DNA control obtained by an asymmetric PCR (in substitution of cDNA obtained by reverse transcription). The highest error rate obtained in one control (9.2 × 10^−5^) was not far from the lowest error rate reported (3.4 × 10^−4^), which may indicate a high background error in their method. Furthermore, they found a large number of C→A and G→T substitutions in their controls that were not considered in their RT error rate determinations.

In the study by Martinez del Río et al. [56], the authors also included a DNA control in their experiments of cDNA barcoding fidelity measurements. The DNA control was produced through a single-cycle PCR using the high-fidelity Q5 DNA polymerase and a plasmid-derived DNA as a template. This DNA control underwent the same preparation and sequencing processes as the cDNAs. When using a threshold of 100%, their control exhibited a very low error rate of 3.67 × 10^−7^, close to a previous estimate of the Q5 DNA polymerase error rate [77]. However, when using a 0% threshold, the error rate was significantly higher (1.36 × 10^−5^), highlighting the significant impact of threshold values on specificity.

Additionally, methods like DUP-SEQ, compared to PRIMER IDs, offer improved specificity due to the creation of two independent consensus sequences: one from the cDNA strand and one from its complementary strand. This dual-consensus approach enhances specificity by identifying and discarding errors that appear in only one strand, as they are treated as artifacts. Schmitt et al. [59] reported that DUP-SEQ achieves a theoretical background error rate as low as 10^−9^. However, this increased specificity comes at the cost of greater methodological complexity. Moreover, the need to amplify and sequence both strands for each cDNA molecule reduces the overall yield of consensus sequences obtained compared to single-strand consensus sequencing methods. This lower efficiency can compromise the sensitivity of the approach, although bioinformatics analyses have been proposed to enhance sensitivity while maintaining the specificity characteristic of duplex sequencing methods [78].

### 5.4. Artifacts Affecting the Mutational Spectra and Hotspot Distribution

Another challenge is the non-uniform distribution of reverse transcription errors along the sequence being studied. For example, both *lacZ* forward mutation assays [79,80] and NGS-based assays [56] have reported a remarkable accumulation of errors at the first positions that are reverse transcribed by the viral polymerase. The inclusion of these positions in the error rate calculations could have a large effect on the obtained values, especially when the consensus sequences are relatively short. This elevated error rate at the initial positions has also been observed with other DNA polymerases [81]. Therefore, if the NGS method includes a PCR (or a rolling circle) amplification step, there could be an accumulation of errors that could contribute to a higher background error rate. Furthermore, analyses of sequences obtained with the Illumina platform have identified a high frequency of mutations at both ends of the sequenced insert. This pattern is characterized by A→C substitutions at the 5′ end and T→G substitutions at the 3′ end [42,82].

Another issue associated with most methods is the inability to exclude errors already present in molecules used as a template for reverse transcription. Since RNA cannot be synthesized with high-fidelity polymerases, this problem becomes more significant when RNA is used as a template instead of DNA. Consequently, when using RNA as a template, the error rates measured after the consensus construction represent the cumulative error of reverse transcription and RNA transcription errors. This phenomenon, designated as “transcriptional threshold”, may attenuate RNA-dependent DNA synthesis fidelity when using high-fidelity RTs [80]. Methods that involve labeling RNA molecules can address this problem, but they come with increased technical difficulty and higher costs. On the other hand, methods like ROLL-SEQ avoid the use of RNA barcodes and allow the discrimination of RNA errors from RT errors. However, RTs with elevated rolling-circle activity are needed to generate long concatemeric cDNA. This might be possible with thermostable intron II RTs that appear to be particularly efficient in copying large transcripts [30,31,32].

### 5.5. Artifacts Due to the Generation of “Offspring Barcodes” and Barcode Duplications

**Figure 7:**
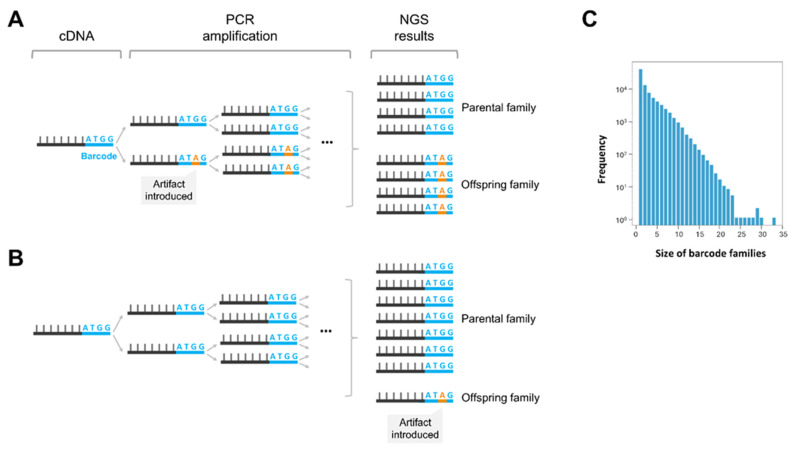
Effect of mutations in barcodes on the generation of offspring barcodes. (**A**) Impact of introducing an artifact in a barcode during the early PCR cycles. (**B**) Impact of introducing an artifact in the later PCR cycles or during sequencing. (**C**) Barcode frequency distribution in a typical NGS experiment. Ordinate values are represented on a logarithmic scale. Adapted from [56].

The generation of “offspring barcodes” or “false families” (i.e., the appearance of artifacts in sequenced barcodes) can be another difficulty associated with the use of barcode-based methods, such as PRIMER IDs, REP-SEQ or ARC-SEQ (Figure 7). This can lead to situations where distinct consensus sequences actually originate from the same cDNA molecule, potentially quantifying the same error twice, or it can lead to a loss of sensitivity if the offspring barcode shares its sequence with another barcode (Figure 6B). Zhou et al. [83] addressed this issue, and based on calculations of maximum frequencies of offspring families, they provided cutoff values that should be used to minimize the counting of offsprings. This value is directly correlated to the number of different barcodes observed after sequencing. However, these calculations were obtained assuming that sequencing and other artifacts have a cumulative error rate of 2% in the sequences. In addition, the election of high cutoffs to avoid offsprings may diminish the robustness of error rate calculations, since the majority of barcodes are often repeated only a couple of times. In fact, it is difficult to obtain barcodes that are repeated. The majority of barcodes in experiments are repeated only once [53,56], which does not allow the construction of consensus sequences. Figure 7C shows the typical distribution of barcode frequencies.

A problem affecting barcode-based methods is the occurrence of repeated UMIs (collisions or tag clashes). This can be attributed to random assignments or because an offspring sequence generates a barcode that already exists in the barcode pool. Collisions may result in underestimation or overestimation of mutation frequency, although, on average, underestimations are more probable according to informatics simulations [84]. To reduce the number of collisions, it is ideal to use a small number of input molecules and a much larger number of barcode combinations. This number is directly proportional to the barcode length. Nevertheless, detecting low error rates requires a high number of input molecules, which can sometimes exceed the available barcode combinations, thereby increasing the likelihood of collisions. In the study by Jabara et al. [49], the authors used barcodes that were only eight nucleotides long, thereby increasing the likelihood of repetition, as pointed out by Sheward et al. [85], after applying formulae related to the well-known birthday paradox and demonstrating the high probability of having repeated barcodes. However, longer barcodes also increase the likelihood of obtaining offspring barcodes and potentially affect error rate measurements [86]. It is also worth noting that even in cases where the probability of having at least one barcode is high, the expected number of collisions may be very small to be able to have an impact in error rate calculations [87]. In Table 1, we show the expected number of collisions for different numbers of barcodes used and for different barcode lengths.

## 6. The Intrinsic Fidelity to Retroviral RTs Calculated by NGS Methods

Forward mutation assays using the M13mp2 *lacZ*α reporter gene [39] have been widely used to determine the DNA-dependent DNA synthesis fidelity of many different RTs. Calculated mutation rates range from 5.8 × 10^−6^ for feline leukemia virus RT to 1.7 × 10^−4^ for prototype primate foamy virus RT [55,80,88,89,90,91,92,93,94,95,96,97]. As discussed above, these are underestimates of the error rate since *lacZ*α-based assays are unable to detect silent mutations, as well as those that do not produce a detectable phenotypic change. In addition, variability in the reported error rates has been observed for the widely studied HIV-1 RT, with values in the range of 6.3 × 10^−5^ to 1.9 × 10^−4^ (reviewed in [9]). The high error rates of HIV-1 RT (around 10 times higher than those of AMV or MLV RTs) have been attributed to the relatively high Mg^2+^ concentration used in the assays. Achuthan et al. [98] showed that the accuracy of purified HIV-1 RT could be increased up to 5 to 7 times in the presence of free Mg^2+^ concentrations similar to those found in lymphocytes (~0.25 mM).

The comparison of error rates obtained with HIV-1 RT in reporter gene-based assays failed to provide consistent results when comparing RNA vs. DNA templates. These assays are technically more difficult to perform if RNA is used as template. However, error rates of 1.9 × 10^−4^ for DNA-dependent DNA synthesis and 2.0 × 10^−4^ for RNA-dependent DNA synthesis were obtained from studies carried out with HIV-1 RT using M13 and *env* V1 as the vector and reporter gene, respectively [99]. The differences were also small for similar assays carried out with the pBluescript plasmid and *lacZ* (1.7 × 10^−4^ and 1.4 × 10^−4^, for DNA and RNA templates, respectively) [100]. However, other studies showed that RNA-dependent DNA synthesis was around three- to five-fold more accurate than DNA-dependent DNA synthesis in reactions catalyzed by HIV-1 RT, in the presence of high concentrations of Mg^2+^ [79,80]. Using forward mutation assays and *lacZ*α as a reporter gene, Sebastián Martín et al. obtained error rates of 2.5 × 10^−5^ to 3.5 × 10^−5^ for RNA-dependent DNA synthesis with different retroviral RTs, including those of HIV-1, MLV and AMV [80].

As discussed earlier, these values are underestimates and include the transcriptional error rate of phage T7 polymerase used in the synthesis of the template RNA. However, these cumulative error rates (resulting from errors in the template RNA, plus errors in the cDNA synthesized by the RT, and any potential changes occurring in the bacteria) were broadly consistent with error rates calculated ex vivo using *lacZ* as reporter gene and estimated at around 3.4 × 10^−5^ per nucleotide and replication cycle in HeLa and CEM cells [101,102] and 1.4 × 10^−5^ in HOS cells [103].

One of the most popular applications of NGS in retrovirology focuses on the detection of minority variants in the viral quasispecies, particularly for the early identification of drug-resistant HIV variants that could hamper the efficacy of antiretroviral therapies (for recent reviews, see Refs. [104,105]). NGS platforms are error-prone, and the use of barcodes or UMIs (originally called Primer IDs) during cDNA synthesis opens the possibility of detecting minority variants present at less than 20% abundance [49,83], as reviewed in Ref. [106]. In these assays, total HIV RNA was obtained from infected patients, and the frequency of mutations was determined with NGS and a cDNA barcoding method. These assays allowed the calculation of error rates for reverse transcription catalyzed by SuperScript III RT while copying two different regions of the HIV-1 *env* gene (corresponding to V1/V2 and C2/V3 regions) and the protease-coding region (Table 2). The calculated overall error rate was around 1.1 × 10^−4^, although the base substitution error rates showed striking variability and ranged from 3.0 × 10^−5^ for env V1/V2 to 1.2 × 10^−4^ for the protease-coding region [83].

SuperScript RTs are engineered MLV RTs that show high thermostability and are frequently used in RT-PCR and other applications [17]. As shown in Table 1, error rates obtained with engineered MLV RTs (e.g., SuperScript II, III and IV; MM4; and ProtoScript II) were in the range of 5.0 × 10^−5^ to 1.8 × 10^−4^ and were not largely affected by the origin of the RNA used in the assays (i.e., natural RNA vs. RNA synthesized with phage RNA polymerase). The differences between wild-type and mutant MLV RTs were relatively small in experiments where enzymes were assayed under the same conditions [54,64]. Potapov et al. [64] also showed that the error rates of AMV and MLV RTs were very small (7.5 × 10^−5^ and 6.3 × 10^−5^, respectively), and both enzymes showed a preference for T→C as the most frequent substitution introduced in the reverse transcription process (Table 2). The substitution T→C was also the most frequent mutation introduced by group-II intron-encoded RTs (e.g., MarathonRT, TGIRT and Induro RT) [31,64]. These enzymes showed cumulative error rates slightly reduced in comparison with SuperScript IV [31].

In contrast, the calculated HIV-1 RT error rates were higher, in agreement with previous reports based on the results provided by forward mutation assays and the M13 *lacZ*α reporter gene. Side-by-side comparisons of HIV-1 RT and a thermostable variant of MLV RT showed a 2.6-fold increase in the cumulative error rate of the HIV-1 enzyme, measured on the *cesD* gene of *Bacillus cereus* [52]. The calculated HIV-1 RT error rate (around 3 × 10^−4^) was obtained from cDNA synthesis reactions carried out in the presence of 5 mM Mg^2+^ and was reduced to 1 × 10^−4^ in 1 mM Mg^2+^ [53]. Similar effects, caused by the reduction in the Mg^2+^ concentration, were also reported by Wang et al. and Martínez del Río et al. using the M13 *lacZ*α and the HIV-1 protease-coding sequences, respectively, as targets [55,56]. The lower error rates reported by Martínez del Río et al. (Table 2) can be attributed to the consensus and threshold values used in their experiments. These conditions were introduced to improve the specificity of the assay and eliminate potential artifacts not discarded during consensus construction. On the other hand, the error background reported by Wang et al. [55] was very high, as determined in their negative controls. Error rate estimates of HIV-1 RT have been generally obtained using in vitro transcribed RNA [52,53,55,56]. Errors made by phage polymerases during transcription can be a source of errors. However, Martínez del Río et al. [56] have shown that the RNA source has little impact on the overall error rate, although the most prevalent mutations differed between both experiments. A detailed analysis of the locations and mutation types obtained in both assays revealed a likely contribution of adenosine deaminases (e.g., ADAR1) in the observed variability, as suggested in other studies [103,107].

Most of the studies described in Table 2 provide cumulative error rates (i.e., those resulting from the combination of errors present in the RNA source and those made by the RT during cDNA synthesis). In some studies, the RNA source was heterogeneous in nature and reflected the variability of the viral quasispecies [49,83]. Methods rendering cumulative error rates are not suitable to identify high-fidelity RTs since errors present in the RNA constitute a transcriptional threshold that represents a basal error rate that blurs any improvement in RT’s accuracy. Therefore, a challenge for these methods is the distinction between transcription and cDNA synthesis errors. Theoretically, this could be achieved using different sets of barcodes for RNA and cDNA molecules, as described by Reid-Bayliss and Loeb, using the ARC-SEQ method [58]. In their study, the authors used in vitro-transcribed RNA from phage m13mp18, as well as total yeast RNA as templates. The estimated error rate for the T7 RNA polymerase was around 3 × 10^−5^, while the error rates derived from transcription in the yeasts were estimated at 4.21 × 10^−5^. ProtoScript RT was used for cDNA synthesis in these experiments, but the reverse transcription error rate was not reported.

Using the REP-SEQ methodology, Gout et al. [57] estimated a transcription error rate of 4.1 × 10^−6^, well below their estimated cDNA synthesis error rate, which was 1.14 × 10^−4^ (for SuperScript II RT). Interestingly, the most common mutation introduced in reverse transcription was G→A, while C→T was the most common single nucleotide polymorphism (SNP) found in the RNA. The transcriptional error rate was calculated for the total RNA obtained from *Caenorhabditis elegans*. Potapov et al. recently reported separate error rates for transcription and cDNA synthesis, using a ROLL-SEQ protocol [65]. The RNA template represents an artificial sequence and was obtained by in vitro transcription with T7 RNA polymerase, and the results were analyzed using Pacific Biosciences Single Molecule Real-Time sequencing. The transcriptional threshold was estimated at 5.3 × 10^−5^, similar to the cDNA synthesis error rate of the Induro RT (5.7 × 10^−5^) (Table 2).

## 7. Conclusions and Future Developments

Understanding the fidelity of RTs, along with other sources of mutations, is essential to understand viral dynamics and plays an important role in the development of new tools for biotechnological applications. The accuracy of these enzymes has an important impact on genetic diversity, plays a critical role in the development of drug resistance and represents a critical challenge in vaccine development [108]. Accuracy is important for single-cell transcriptomics, where the detection and precise characterization of low-abundance RNAs are critical [17,109]. RTs with increased accuracy could also be valuable in synthetic biology for long-term nucleic acid data storage that uses naturally nuclease-resistant, chemically modified nucleic acids [110].

Finally, there are biotechnological applications of RTs where low fidelity is sought. One example is the detection of modified ribonucleotides, where having a high error rate at those positions is a desirable feature [111,112,113]. The use of NGS for the detection of RNA modifications has opened the field of epitranscriptomics [114], where the RT error rates could be exploited for the development of new methods to identify modified bases. NGS-based methods using appropriate RTs could avoid the use of antibodies directed against modified bases that often produce inaccurate results. Typically, the “PRIMER IDs” method (Figure 4A) is used to measure errors introduced by the RT when reverse transcribing an RNA template. Modifications are detected as RT signatures (e.g., mismatches, indels or RT stops) that arise when Watson-Crick pairing is disrupted, such as with N^1^-methyladenosine (m1A) [115]. However, certain modifications such as N^6^-methyladenosine (m6A), are categorized as “RT-silent”, and thus pose a greater detection challenge. For this type of modifications, the Watson–Crick pairing is usually not affected and the information about the base modification is erased during the reverse transcription step [116]. However, strategies exist to enhance the appearance of RT signatures, either by engineering the RT [111] or by altering the modifications to induce distortions [117].

Still, the possibility of obtaining more faithful RTs represents an important challenge that cannot be properly addressed without reducing the transcriptional threshold. Obtaining more faithful RNA polymerases could be important to manufacture high-quality mRNA, particularly relevant in vaccine development.

On the other hand, NGS is increasingly used for HIV drug resistance genotyping due to its enhanced sensitivity compared to classical Sanger sequencing methods. However, much of the observed variability in NGS sequences may reflect PCR error rates, rather than authentic mutations. Empirical thresholds for reporting drug resistance mutations detected by NGS are usually between 0.5 and 5% [118,119]. However, Tzou et al. [120] have shown that single genome sequencing (involving the use of barcodes and eliminating most of the PCR errors) could detect highly unusual mutations in the protease-, RT- and integrase-coding regions in proportions ranging from 3.9 to 6.5%, suggesting that the thresholds to be used for HIV drug resistance genotyping should be higher. In line with this proposal, comparative studies carried out in different laboratories have shown that the best agreement between NGS and Sanger sequencing methods was obtained when minority variants represented more than 20% of the NGS variation observed at one position [121], although other studies suggested that lower thresholds could be valid for specific resistance mutations (e.g., for those associated with resistance to non-nucleoside RT inhibitors) [122]. Still, the detection of minority variants is limited by errors made by the RT used in cDNA synthesis, prior to PCR amplification, and the bioinformatics pipelines used in the analysis.

Taken together, measuring transcriptional error rates, as well as designing reliable methods to reduce cDNA synthesis errors is still challenging, requiring laborious and expensive methodologies. Further developments in NGS affecting accuracy, reliability and cost will be necessary to address these issues.

## Figures and Tables

**Table 1:** Expected collisions for degenerated barcodes of 8-, 10-, 12-, 14- and 16-nt. Calculations were determined using the “birthday problem” formula indicated below, assuming different numbers of barcodes ^1^.

Number of Barcodes (*n*)	Expected Number of Collisions				
	8-nt UMI	10-nt UMI	12-nt UMI	14-nt UMI	16-nt UMI
1000	15.13	0.95	<0.01	<0.01	<0.01
2000	60.08	3.81	0.24	0.02	<0.01
5000	367.21	23.78	1.49	0.09	<0.01
10,000	1415.05	94.91	5.96	0.37	0.02
20,000	5259.93	377.84	23.83	1.49	0.09
50,000	26,685.09	2328.19	148.79	9.31	0.58
100,000	78,256.93	9096.03	594.27	37.25	2.33
500,000	499,757.03	189,627.97	14,681.28	930.45	58.20
1,000,000	999,999.76	614,677.19	57,863.01	3718.36	232.80
2,000,000	2,000,000.00	1,703,052.94	224,755.88	14,845.78	931.11
10,000,000	10,000,000.00	9,999,278.49	4,490,142.91	365,675.47	23,255.98

^1^ Calculations shown in the table were carried out using the classical “birthday problem” formulation. Instead of individuals, we used barcodes (*n*), and instead of the 365 possible birthdays, we considered the different combinations for a barcode of a certain length (c). The expected number of barcode “collisions”, i.e., the number of barcodes sharing the same sequence in a given experiment (or data collection), is given by the following equation: E (number of barcodes with shared sequences) = *n* (1 − (1 − (1/c))*^n^*^−1^).

**Table 2:** RT error rates calculated by NGS for RNA-dependent DNA synthesis, as determined in different studies ^1^.

Study (Ref.)	RNA Source (Target Sequence)	RTs	RT Error Rates	Most Common SNP	Method	Consensus Cutoff	Threshold (%)
Jabara et al. [49]	Human plasma samples HIV-infected subject (HIV-1 PR-coding sequence)	**SuperScript III**	7.05 × 10^−5^	-	cDNA barcoding	3	0
Zhou et al. [83]	HIV-1 RNA from 8E5/LAV infected cells (HIV-1 *env* C2/V3 and V1/V2, HIV-1 PR)	**SuperScript III**	1.1 × 10^−4^	A→G	cDNA barcoding	Variable (to avoid offsprings)	0
Gout et al. [57]	*Caenorhabditis elegans*	**SuperScript II**	1.14 × 10^−4^	G→A	REP-SEQ	2–3	100
Ellefson et al. [51]	Human heart total RNA (*HSPCB* gene)	**MMLV**	1.1 × 10^−4^	G→A	cDNA barcoding	3	66.6
		RTx	3.7 × 10^−5^	G→T			
		RTx (exo-)	1.0 × 10^−4^	A→G			
Potapov et al. [64]	In vitro transcription using T7 RNA polymerase (artificial sequence)	**MMLV**	6.3 × 10^−5^	T→C	SMRT-SEQ	-	-
		**ProtoScript II**	5.6 × 10^−5^	T→C			
		**AMV**	7.5 × 10^−5^	T→C			
		Bst	1.8 × 10^−4^	T→C			
Zhao et al. [31]	RepA D3 RNA (a 1.6-kb mouse lncRNA)	**SuperScript IV**	1.8 × 10^−4^	-	cDNA barcoding	3	100
		MarathonRT	9.9 × 10^−5^	T→C			
		TGIRT	1.3 × 10^−4^	T→C			
Potapov et al. [65]	In vitro transcription using T7 RNA polymerase (artificial sequence)	Induro RT	1.1 × 10^−4^	A→G	ROLL-SEQ	3	100
		L1-RT	2.8 × 10^−4^	T→C			
		Fbu	1.8 × 10^−4^	T→C			
Houlihan et al. [54]	Human total RNA (GAPDH mRNA)	**MMLV**	9.77 × 10^−5^	C→T	cDNA barcoding	3	66.6
		**SuperScript III**	5.01 × 10^−5^	C→T			
		**ProtoScript II**	7.66 × 10^−5^	T→A			
		RTx	9.60 × 10^−4^	A→G			
		RT521K	2.51 × 10^−3^	C→A			
		RT-TR	1.22 × 10^−5^	G→A			
		RT-H11	1.08 × 10^−5^	G→A			
Yasukawa et al. [52]	In vitro transcription using T7 RNA poly-merase (*cesD, B. cereus*)	**HIV-1**	2.6 × 10^−4^	A→G	cDNA barcoding	5	100
		**MM4**	1.0 × 10^−4^	C→A			
		RTx	7.5 × 10^−5^	C→A			
Okano et al. [53]	In vitro transcription using T7 RNA poly-merase (*cesD, B. cereus*)	**HIV-1**	1 × 10^−4^ (1 mM Mg^2+^)	-	cDNA barcoding	5	100
			3 × 10^−4^ (5 mM Mg^2+^)				
Wang et al. [55]	In vitro transcription using T3 RNA polymerase (*lacZ*α)	**HIV-1**	3.05 × 10^−4^ (0.5 mM Mg^2+^)	C→T and G→A	cDNA barcoding	5	100
			1.12 × 10^−3^ (6 mM Mg^2+^)				
Martínez del Río et al. [56]	In vitro transcription using T7 RNA polymerase (HIV-1 PR-coding sequence)	**HIV** **-1**	9.0 × 10^−6 -^ (0.5 mM Mg^2+^)	A→G	cDNA barcoding	4	75
			7.3 × 10^−5^ (3 mM Mg^2+^)	G→A			
	HIV RNA from 8E5/LAV infected cell line (HIV-1 PR-coding sequence)		1.8 × 10^−5^ (0.5 mM Mg^2+^)	C→T			
			7.4 × 10^−5^ (3 mM Mg^2+^)	C→T			

^1^ RT sources are AMV, avian myeloblastosis virus; Bst, Bst 2.0 and Bst 3.0 DNA polymerases; MMLV, Moloney murine leukemia virus; MM4 is a thermostable MLV RT containing amino acid substitutions E286R/E302K/L435R/D524A; RT521K is a replicative DNA polymerase from *Thermococcus gorgonarius* that possesses RT activity; RT-TR and RT-H11 are high-fidelity variants of RT521K that contain amino acid substitutions P410T/S411R and R406P/L408A/P410T/S411R/I412F, respectively; RTx is a DNA polymerase from *Thermococcus kodakarensis*; RTx(exo) is the proofreading-deficient *T. kodakarensis* DNA polymerase; Superscript II, III and IV are proprietary thermostable MLV RT variants; MarathonRT and TGIRT are group-II intron RTs from *Eubacterium rectale* and *Geobacillus stearothermophilus*, respectively; Induro RT is a commercial group-II intron-encoded RT marketed by New England Biolabs; L1-RT is a human LINE-1 RT (a non-LTR RT) (LINE: long interspersed nuclear element); and FBu is an RT encoded by the intestinal fluke *Fasciolopsis buski* R2 non-LTR retrotransposon. RT error rates reported in Houlihan et al. [54] correspond to median values. Retroviral RTs are indicated in bold.

## Data Availability

Not applicable.
